# Early detection of breast cancer through the diagnosis of Nipple Aspirate Fluid (NAF)

**DOI:** 10.1186/s12014-024-09495-4

**Published:** 2024-06-28

**Authors:** Abhishek Pant, Ashish. P. Anjankar, Sandesh Shende, Archana Dhok, Roshan Kumar Jha, Anjali Vagga Manglaram

**Affiliations:** Department of Biochemistry, Datta Meghe Institute of Higher Education and Research, Wardha Sawangi Meghe, India

**Keywords:** Nipple aspirate fluid (NAF), Breast cancer, Biomarkers, Liquid Biopsy

## Abstract

The development of breast cancer has been mainly reported in women who have reached the post-menopausal stage; therefore, it is the primary factor responsible for death amongst postmenopausal women. However, if treated on time it has shown a survival rate of 20 years in about two-thirds of women. Cases of breast cancer have also been reported in younger women and the leading cause in them is their lifestyle pattern or they may be carriers of high penetrance mutated genes. Premenopausal women who have breast cancer have been diagnosed with aggressive build-up of tumors and are therefore at more risk of loss of life. Mammography is an effective way to test for breast cancer in women after menopause but is not so effective for premenopausal women or younger females. Imaging techniques like contrast-enhanced MRI can up to some extent indicate the presence of a tumor but it cannot adequately differentiate between benign and malignant tumors. Although the ‘omics’ strategies continuing for the last 20 years have been helpful at the molecular level in enabling the characteristics and proper understanding of such tumors over long-term longitudinal monitoring. Classification, diagnosis, and prediction of the outcomes have been made through tissue and serum biomarkers but these also fail to diagnose the disease at an early stage. Considerably there is no adequate detection technique present globally that can help early detection and provide adequate specificity, safety, sensitivity, and convenience for the younger and premenopausal women, thereby it becomes necessary to take early measures and build efficient tools and techniques for the same. Through biopsies of nipple aspirate fluid (NAF) biomarker profiling can be performed. It is a naturally secreted fluid from the cells of epithelium found in the breast. Nowadays, home-based liquid biopsy collection kits are also available through which a routine check on breast health can be performed with the help of NAF. Herein, we will review the biomarker screening liquid biopsy, and the new emerging technologies for the examination of cancer at an early stage, especially in premenopausal women.

## Introduction

The changing lifestyle these days has not only brought new changes in people’s lives but has also increased the chances of various deadly diseases. One such deadly disease occurring in females is breast cancer. It is in due course of time a global health problem that is largely affecting females and up to some extent males. According to 2020 reports, in the last five years, women affected by cancer were around 7.8 million [1]. It has become the major reason for mortality in women in the age group of 40 to 59 years, the premenopausal women based in Central, America, Africa, and South America are more prone to be affected with breast cancer [2]. The Surveillance, Epidemiology, and End Results (SEER) analysis record shows victims aged under 40 lose their lives more from cancer than those over 40 years of age although the underaged women count was less than 6.4% [3]. The awareness programs and diagnosis techniques fall short of providing adequate and effective information on prevalent breast cancers which is also the reason for the increasing number of deaths. Several risks and factors associated with breast cancer are listed below-.

1. Lifestyle patterns including smoking, alcohol consumption, obesity, food intake, and physical fitness [4].
2. Genetic predisposition [5].
3. Demographic factors including age, sex, socioeconomic conditions, and geographical conditions [6].
4. Reproductive factors including menarche, menopause, parity, lactation [7].
5. Environmental factors including infectious agents, chemical exposures, and drugs [8].
6. Systemic factors such as epigenetics [9].

The environmental, lifestyle, and reproductive parameters causing breast cancers indicate that the build-up of tumors takes a long time and is expressed at a later stage of life. Genetic predisposition leading to breast cancer can only be identified at initial stages through the study of pedigree and can be assured by the test for predictive mutations related to genes having a high rate of penetration such as TP53, PTEN, BRCA1, BRCA2 [5]. Amongst all the women having breast cancer, 5–10% of the women exposed to genetic mutations had chances for cancer development. However, post-menopausal women counter it at a large rate [10]. The genomic technologies prevailing over the last 20 years have been greatly helpful in understanding the hierarchy of the disease. It provides hints for both the progression of the disease and for the clinical directions that could help in the treatment and predictions of the outcome. To date five main types of phenotypic characteristics of breast cancer have been observed which include Luminal A, Luminal B, Her2, Claudin-low, and basal character, further stratification can be anticipated [11]. Concerning proteins of cell surface expression and repression, characterization of phenotypes is made which comprises -.

Progesterone Receptor (PR).

Estrogen Receptor (ER).

Human Epidermal Growth Factor Receptor (HER2).

The above-mentioned phenotypic characteristics also correlate with the development of normal mammary cells [12]. Several triple-negative breast cancers (TNBC) have been shown by the Claudin-low phenotype originating from the breast stem and the basal-like character originating from progenitor cells. It is also related to obesity in premenopausal women [13] and to BRCA1 and BRCA2-induced cancer [14]. TNBC has a poor prognosis, it can re-occur frequently and thereby it has no accurate treatment the survival rates decline. The extensive etiological and epidemiological studies have helped in the long term to identify breast cancer risks but none of the methods have been developed to determine at which stage the cancer is likely to occur. However, certain screening procedures can be employed in women after menopause or in those in reproductive years which can help monitor the occurrence of breast cancer. Table 1 illustrates various screening techniques to determine breast cancer along with their advantages and disadvantages. The screening procedures fail to explain how to prevent breast cancer, therefore it is essential to build new standards and techniques to detect and treat breast cancer. Below are some of the methods through which breast cancer can be analyzed.

### Detection techniques of breast cancer

Few approaches are required to be made for early detection which will aid in less drastic means of intervention. Some of the prevalent screening techniques are-.

### Self - examination

Active surveillance can be done by the females themselves to check for their breast health. Self-examination can help discover the natural changes taking place from time to time and about 50% of detection of the build-up of tumors or other breast-related abnormalities without the aid of any specialist or professional health assistance [15]. But the fact is that the rate of self-examination is very low, especially in areas where literacy and awareness are less [16].

### Mammography

Mammography is yet another common approach that enables screening of breast cancer. Every year, around 39 million women in the US between the age group of 50 to 74 years are examined and every 2–3 years, women of age between 50 and 70 undergo breast screening. National screening programs are held by the government for the detection of the build-up of tumors and for premenopausal women annual screening programs with magnetic resonance imaging (MRI) and mammography are held which help in certain risk-benefit decisions. Mammography is likely to vary from 100 to 40% based on the tissue composition [17, 18] while on the positive interaction with the hypothesis of disease, it can be up to 50%. The screening tests also have some limitations such as false positive rates which have high call-back rates, radiation exposure, anxiety in patients at the time of screening, and unnecessary biopsies that result in increased expenditure [19]. Particularly in women of age group under 50, the mammography is less sensitive when compared to postmenopausal women [20]. However, the full field digital mammography with enhanced resolution techniques and digitization has made it more versatile and a bit accurate in screening practices but it still has some cons thereby it needs some more inputs to help detect the amount of mass in the breast [21, 22]. Many times the tumors show metastasis even before their detection, moreover, the mammography technique also fails to differentiate between benign micro-calcifications related to acute risk DCIS (Ductal Carcinoma in situ) and chronic risk DCIS that can lead to malignant invasive tumors [23]. These when supported by histopathological screening, may cause overdiagnosis followed by treatment of those affected with DCIS [24]. A false positive diagnosis enables surgery in which the whole lump or breast is removed. Exposure to certain radiations may lead to several heart diseases induced by cell walls or breast radiotherapy [17]. The breast density is related to tissue composition which includes collagen fibers, epithelial cells, glandular region, certain genetic influences, and hormonal regulations. The screening techniques in younger women fail to analyze the breast tissue density whereas in postmenopausal women the breast tissue filled with fat or ductal atrophy gives an accurate diagnosis [25].

### Digital breast tomosynthesis (DBT)

It is a novel 3D method that uses an X-ray beam in an arc that provides a 3D structure of the affected tissue. DBT has shown better results when compared to digital mammography. It also has the same limitations as the mammography technique in that it fails to recognize breast density. The DBT shows about 90% sensitivity and 79% specificity in contrast to mammography which shows about 89% sensitivity and 72% specificity [26]. Below mentioned are some other imaging techniques that help in breast cancer detection.

**Ultrasonography**- is a technique that uses linear transducers of high resolution. It is easily accessible; the cost of mammography and ultrasonography is almost the same but the latter provides slightly improved results. However, it has low specificity and, a low beneficial predictive score, and it also fails to analyze the tissue density [27].

**Contrast-enhanced MRI** – a study was performed and the MRI proved to be 94% sensitive whereas very few cases of cancer were reported when compared to digital mammography [28]. The MRI showed relatively low specificity and needed more cost and time to operate.

**Positron Emission Tomography (PET)-** this technique in addition to computer tomography (PER-CT) utilizes the glucose analog- gamma–radiated emitting fluorine − 18 fluorodeoxyglucose (FDG). This injection into the veins accumulates in the regions where the density of tissue is high such as in regions of tumors analyzed through PET-CT x-ray scanner [29]. This technique has proved to be about 71 to 90% sensitive and provided much more accurate results with breast size when compared with digital mammography [30]. However, the use of PET-CT is more in assisting surgery to remove diseased tissue rather than identifying the occurrence of breast cancer. MRI techniques or ultrasound is also used in wave elastography technique to check for tissue stiffness which indicates the presence of a tumor as the breast stiffness is different in normal cases. Some more specificity and sensitivity are provided by shear wave elastography which makes use of the force of acoustic rays produced by an ultrasound beam.

**Electrical Impedance**- it is yet another method being employed that can scan for breast tumors, especially in younger women having dense breast tissue. It shows lower electrical impedance when compared to the normal surrounding tumor in the case of a malignant tumor [31].

Some modern techniques have also been developed that can record the variations in circadian rhythms and analyze the recorded data but also generate several false positive rates. Out of all these detection techniques, mammography is the most reliable tool to scan for breast cancer.

**Table 1:** Indicating different screening techniques for identification of breast cancer

Techniques	Identification	Percent Sensitivity	Percent Specificity	Benefits	Drawbacks
Self- examination [15]	Diagnosis of Tumor	53.90	54–59	an inexpensive and flexible way of detection reduces mortality	Few breast lumps miss out, causing unnecessary distress
Mammography [20]	Diagnosis of Tumor	73–86	88–93	Low-cost, portable, contrast reagent not needed	Inconvenient, penetration only to a limited depth, hard, exposed to radiation, incorrect positive - negative results.
Contrast enhancement ultrasound [27]	Detection Tumor and depiction	61.40	82	Portable, cost-effective	vascular system can only be defined by contrast agents
Magnetic Resonance Imaging (MRI) [28]	Tumor depiction	77–99	81–99	Quantifies tumor perfusion and tumor permeability inside the capillary	restricted capacity has a contrasting design, limited for magnetic atom
Positron Emission Tomography (PET) [29]	Diagnosis response and its evaluation along with a depiction	64–96	73–99	probes involving molecular imaging tracing imaging without perturbing biological systems on a wide range	spatial resolution is limited and uses non-contrasting computed tomography, may also exposure to radiation
Histopathology [24]	Diagnosis and Tumor Classification	90	88	Differentiates between benign tumor and malignant tumor	Causes pain as it involves surgery, infection, and bleeding. This can lead to overdiagnosis and treatment.

**Fig. 1:**
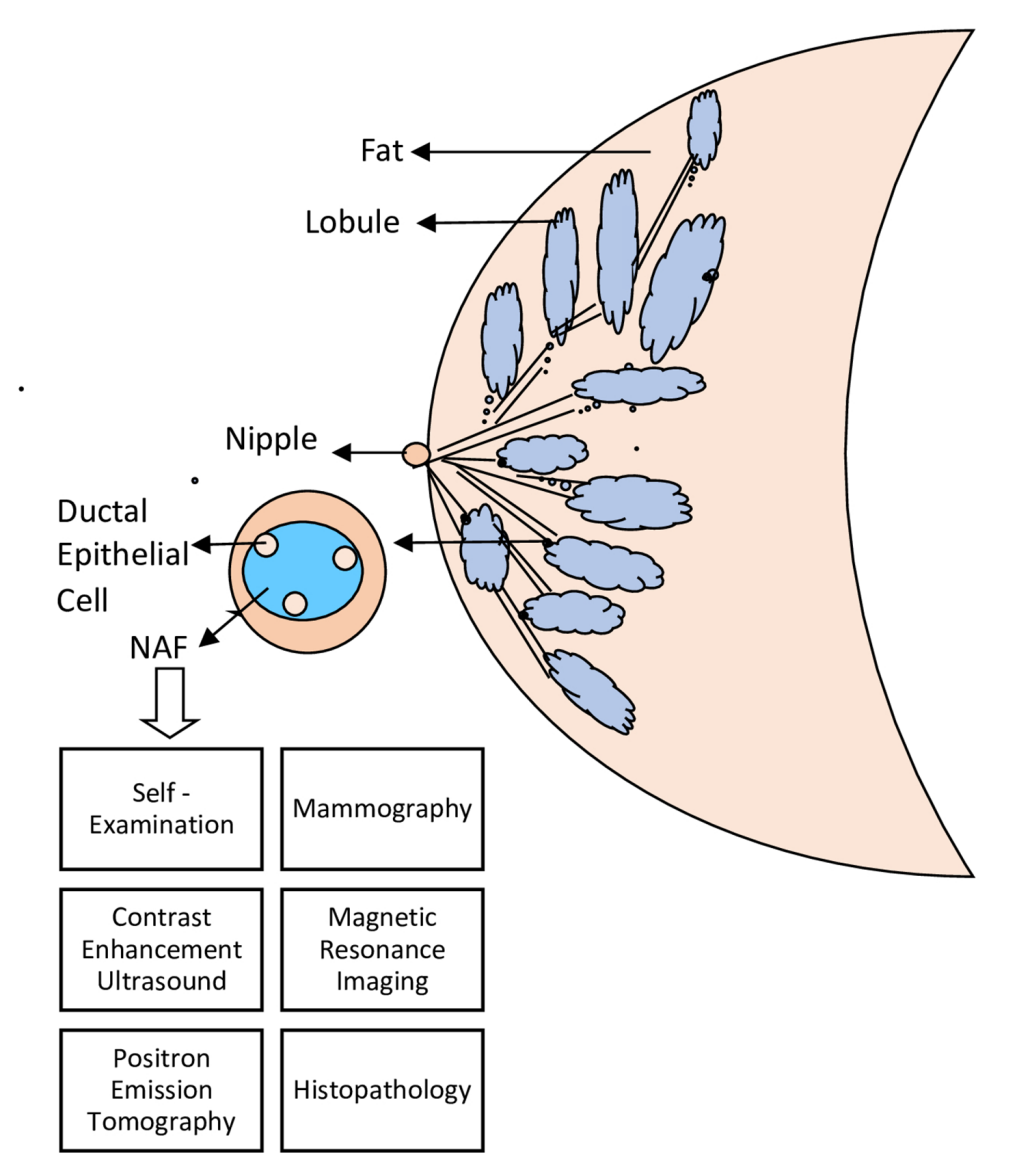
Representing different methods of detection of Breast Cancer

### Proteomics

Proteomics involves the study of proteins on a large scale encompassing the identification of proteins, their oncology, pathways involved in them, protein-to-protein interaction, their functional analysis, quantification, identification of protein subgroups like kinases, phosphorylated and secreted proteins, exosomal proteins and proteases.

### Current proteomic techniques used in the study of breast cancer

Several proteomic studies have been made to study all types of cancers. Below are some of the techniques used in the study of breast cancer.

### Plasma proteomics

Plasma is an easily available resource that can help discover biomarkers. However, the complexity and high concentrations of proteins in plasma may pose several difficulties in MS-based proteomic analysis. However, the circulating proteins can be characterized by the studies available on MS-based proteomics of plasma [98].

### Tissue proteomics

The tissue samples are an effective and attractive source for the study of biomarkers that can help identify proteins effective in clinical assessment. A very small portion of tumor tissues obtained through surgical specimens and biopsies can be used for protein extraction. Moreover, it helps in identifying the diversity of proteins contained within.

Certain information related to prognosis, treatment, and response can be obtained through breast cancer subtyping by the use of gene expression profiling and immunohistochemistry (IHC). Several new protein biomarkers having distinct gene aberrations may be identified through proteomic analysis that may further serve to refine the present molecular characterization. These days proteomic technologies allow an analysis of protein expressions in many samples [99].

### Biomarkers

Biomarkers are yet another effective way of detection. There has been extensive research on biomarkers that can help detect breast cancer. Although some other genomic studies have accelerated the process and helped in the medication of Estrogen Receptor/Progesterone Receptor breast cancer including microarray-based techniques that utilize regular prognostic multigene signature screening of 70 gene MammaPrint, metastatic prognosis, chemotherapy [32], Celera 14 gene is applicable for metastasis of tumors [33]. Gene signature Oncotype DX 21 is used for the calculation of chances of re-occurrence [34], and the signature 76-gene Veridex helps in the therapy of tamoxifen [35]. Certain proteins and metabolites have been identified which were found to be more in malignant tissues but they are present in very diluted form in plasma and urine so cannot be detected. Effective measures are required to segregate the excess proteins such as immunoglobins or albumins present in plasma before detection which may again cause reduced sensitivity, thereby these could not specifically help in the detection of breast cancer [36]. The Committee of the American Society of Clinical Oncology in a review brought up in 2007 failed to present any method for blood level extent of CA 15 − 3 and CA 27.29 detection, decisions on metastasis, or circulating extracellular HER2 for breast cancer detection [37]. Tissue biopsies are performed by looking at tumor-associated biomarkers, where their concentration is found to be higher, although they are not that practical certain biomarkers used in tissues include HER2, Estrogen Receptor, Progesterone Receptor, urokinase – type plasminogen activator (uPA), Plasminogen activator inhibitor 1 (PAI-1) through ELISA Test or immunohistochemistry. Therefore, the search continues to find techniques that are cost-effective, safe, simple, sensitive, and accurate for the earliest detection of the presence of breast cancer. Table 2 illustrates a quick summary of various approaches for the detection of breast cancer.

### Liquid biopsies of breast (biofluid biopsies)

The best bioliquid for detection are those that lie in close relation to the diseased tissue and can be readily available such as urine in case of bladder or kidney cancer, or saliva in case of oral cancer. Several methods have been developed and brought into use for accessing the ductal liquids associated with the cells from which the majority of breast cancers originate. One such fluid is the Nipple Aspirate Fluid (NAF) which can help in the screening for breast cancer. Several other fluids secreted from breasts have been reviewed and studied moreover their proteomics have been discussed with great interest, some of them are mentioned below-.

### Milk and colostrum

Several studies on the proteomics of milk have been made that were done to study the functional characteristics of milk proteins, and about 1600 proteins have been identified in a recent study [38, 39]. Colostrum is a thick yellow milk secreted two to three days before lactation. It is enriched with antibodies that boost the immune system of the infants. About a hundred samples were studied by the use of (LC-MS) 2D liquid chromatography-mass spectrometry in which 151 proteins were analyzed after immunodepleting to report the most found proteins that comprised 83 proteins in colostrum [40]. The secretion of milk and colostrum is sufficient for analysis but the reproductive period causes a reduction in its production and cannot prominently help indicate the proliferation of breast cancer. Therefore, the proteomic studies of milk and colostrum in association with the development of breast cancer have contributed very little, as very few numbers of cases women developing both breast cancer and the post-natal phase have been reported. Nevertheless, a study was found to be successful in which proteins were diagnosed as present in milk secreted by women having breast cancer [41].

### Breast cyst fluid

A relation between cystic breast disease and cancer can be derived from certain epidemiological and prospective studies [42]. Breast fluids provide many biomarkers by application of unsupervised and supervised cluster analysis[102]. Breast cysts are of two types characterized in terms of certain morphological and cell characteristics (type 1 or apocrine cyst and type 2 cyst). The apocrine cyst has a higher potassium and sodium ratio, and type 2 cysts are highly connected to cancers in the breast region [43]. Various observations have been made on breast cyst fluid that showed about 81 proteins in it. The majority of the proteins found were prolactin, albumin, inducible protein, apolipoprotein D, and Zn alpha 2 glycoproteins. Additionally, through the ELISA test it was found that the nipple fluid contains DJ-1 protein, and its high level is found to be associated with the presence of cancer cells. A study using 2D gel electrophoresis was performed on macrocyst fluid of the apocrine and it was recognized for 3 hydroxymethylglutaryl- CoA synthase and 15-hydroxyprostaglandin dehydrogenase that was related to cyst and tumor tissue as it was missing in tissue present in the normal areas [42].

### Ductal Lavage (DL)

It is a method that can help analyze greater breast cancer chances moreover it helps in scanning for the malign abrasion in breast epithelium through non-surgical methods. Ductal lavage involves the infusion of saline solution through a microcatheter into the ducts. It is then infused with the help of suction to obtain cells of the duct lining. The cell count collected by the use of this procedure is far more than the cells collected from nipple aspiration [44]. Cells from 31 women having breast cancer were collected and analyzed for atypical cytology and only 13% gave a significant positive result [45]. Another study on 30 victims was done predicting 23.3% of women victims affected with atypical lavage cytopathology, all of them depicted usual breast mammogram screening which showed improved sensitivity [46]. The cells isolated from ductal lavage can be used in molecular biology. QM – PCR (Multiplex methylation-specific polymerase chain reaction) has been applied in cumulative gene promoter hypermethylation in numerous genes, these are certain biomarkers that help to scan for the presence of breast cancer moreover, they can enhance sensitivity twice for analysis of cancer-causing cells in contrast with cytology [47]. An approach through ductal lavage was made to identify tamoxifen action-related biomarkers which showed no notable variations at cell or molecular level in patients [48]. Ductal aspiration was done by advanced approach through a collection of multiple aliquots that showed an increase in the cell recovery in which 45 out of 50 patients showed more than 1000 cells and 50% of those produced more than 20,000 cells having 80 to 100% purity in epithelial cells through which RNA, miRNA and genomic DNA specimens could be detected. Presently, the observations on a qualitative basis of the molecular profiles can only be recorded [49]. More than 700 miRNAs were identified from ductal lavage of patients affected with unilateral cancer. Among these, 17 patients were found to be affected with different tumors while the normal samples were also found to be related to the processes of occurrence of tumor and signaling pathways for invasion and metastasis [50]. About 43 women having breast cancer were examined and the metabolomic profiles were obtained from their ductal lavage [51]. With a QTOF mass spectrometer detection was done on positive and negative ion mode that showed 2098 compounds from which a signature of 21 metabolites was determined. The metabolites included N-linoleoyl taurine, N-acetyl DL tryptophan, trans-2-dodecenoylcarnitine, and some isoforms of a phospholipid, in the detection of breast cancer, they showed an ROC curve with a sensitivity of 90.7%. ROC or Receiver Operating Characteristic curve is a display of the correct and incorrect positive rate in the form of a graph. While detecting breast cancer in asymptomatic persons, the ROC curve shows a low false positive rate, thereby proving itself to be an effective biomarker for the detection of breast cancer [101]

### NAF

Ductal Lavage also has certain limitations, it can cause discomfort, flushing of ducts, diluted protein components, and can also reduce profiling of biomarker sensitivity.

### Random Peri – areolar fine needle aspiration (RPFNA)

It was brought up in 1998 By Dr. Carol Fabian, it provides details about the breasts of asymptomatic women by breast cell sampling [52]. The cell yield in RPFNA lies between 72 and 85% which is far more than ductal lavage hence, can be performed in several women [53]. 1% lidocaine is given as anesthesia in the breast and thereafter aspirations up to five needles are given in the breast region on the lateral side while four needle aspirations are given in the center. The aspirated fluid extracted contains cells of the epidermis, adipose, immune, and stromal regions [52]. A study performed on 480 women on a family pedigree basis, prior analysis, and biopsy at a precancerous stage revealed the fact that RPFNA improved the analysis of cytological atypia that is at a high risk linked to causing breast cancer in women individuals [52]. 20 of them gave a confirmatory response for the occurrence of breast cancer after 45 months proving that the diagnosis was done by it at a very early stage. A chemoprevention study for the examination of alpha-difluoromethylornithine (DFMO) in women makes use of RPFNA which is at a greater risk. Still, their cytology showed no change while the other molecular markers based on RPFNA like the ones expressing growing antigen of the cell, or expression of growth factor receptor of the epidermal region also showed no change [54]. A study on the proteomic microarray was performed that showed around 60 phosphoproteins that could be analyzed in triplicates ranging from 5000 to 10,000 micro-dissected RPFNA epithelium through which signaling pathways could be tracked to analyze the molecular changes taking place in mammary cancer development. RPFNA also has some limitations, like there may be variations in cell populations that are being tested for particular molecular markers. Moreover, there may be difficulties in obtaining the samples. There may also be difficulty in reproducing this method as it involves repeated input of materials for detection in high-risk women [55].

### Nipple aspirate fluid (NAF)

Women lacking lactation also release very little volumes of fluids that are released into the ducts of the breast [56]. Herein discussion is made on the fluids secreted from nipples collected using massage pumps or using passive discharge. These help in identifying them from invasive methods such as RPFNA or Ductal Lavage. The fluid that moves through the ducts and ampullae via the alveolar glands present in the breast and thereafter moves to the lymphatic ducts and then finally into the blood [57]. Usually, the breast fluid does not come out of the nipple as the ducts are blocked by certain secretions that are viscous or dry or due to the contracting bands of the visceral muscles and keratinized epithelial cells [57]. An equilibrium is maintained between the fluid secretion and re-absorption to keep the breast physiology in equilibrium. The secretion of nipple aspirate fluid depends on many factors such as age, early menarche, ethnicity, lactation history, and consumption of fat or lactose in high amounts [58]. Wax secreted in the ear and NAF both are secreted from the modified apocrine glands. Ear wax is secreted from the ceruminous apocrine gland while NAF is from the mammary apocrine gland. Therefore, women with ear wax secrete a higher volume of NAF than those with dry ear wax [59]. The Pre-menopausal women aged between 30 and 50 years having experience of lactation and those with early menarche secrete higher NAF than those women having no children [58] An analysis of women with pre-menopause aged under 25 to 49 belonging to Asia produced less NAF when compared with women of America [37].

Collection of NAF is possible by altering success rates depending on the procedures and the theorist. To acquire NAF samples breast pumps of electronic and manual origin are utilized in lactation, massage, warming, etc [60]. The oxytocin nasal spray has a widespread role in the secretion of ductal fluids as it increases collection in 95% of patients or volunteers [61]. NAF provides a large number of data related to molecules and cells. A study showed through the proliferation of epithelial disease that breast cancers can be detected easily [62]. This study shows that the exfoliation of the epithelium has increased in the ductal lining and lobules when they grow and multiply using hyperplasia, carcinoma at the same site, and invasive carcinoma as the disease progresses [63]. The cells that are released increase in number and changes were observed in morphology including irregular nuclei, rough cisternae, and fully-developed Golgi apparatus linked to the synthesis of nucleic acid protein [64]. The nipple fluid comprises of few exogenous components including nicotine, cotinine from cigarette smoking, and other endogenous matter including proteins, fatty acids, sterols, lactose, and some hormones like estrogens, progesterone, androgens [56]. The NAF color ranges from brown to blood red, to black, green, shades of yellow, and white [65] which is linked to estrone, estradiol, cholesterol, cholesterol epoxides, and lipid peroxidase concentrations [66]. NAF in its observations has been found to have microbes, the microbes of the duct were found to be different from the skin of the nipple and areola [67]. A comparative study was performed on 6 women having breast cancer with 6 healthy women and differences in a certain general incidence point had a major contribution to breast cancer. The different colors in NAF are highly used as an epidemiological indicator rather than as an indicator for the chances of occurrence of breast cancer. As per the analysis, women having brown or blood-like nipple secretion showed a higher rate of occurrence of breast cancer than those having creamy, yellow, or green discharge [68]. One more study was done related to NAF wherein 327 women were analyzed and the results showed that the frequency of red or brown color enhanced as the cells progressed from pre-cancer stage to post-cancer stage while the biopsy on surgery showed more impact on NAF color than needle biopsy [69]. However, the different collection methods of Nipple Aspirate Fluid also have some disadvantages –.

1. The massage pumps may cause pain, redness, and surface skin lacerations.
2. Ductal Lavage is an expensive and time-consuming method and is not readily available.
3. The oxytocin nasal spray is a drug therefore needs to be administered only in a medical setting.

Secretion of NAF, nutritional value, and level of estrogen are all indicators of breast cancer. 1496 people took part in a study, out of which 1347 were white women and the rest were Asian women, the study showed a strong link between the high fat and secretion of NAF among the age group of 30 to 44 years [70]. High fat consumption causes obesity which can in turn relate to NAF production and composition that helped in the prevention and prediction of breast cancer [71]. A contrary result was obtained when a relation was thought to be established between lactose and soy intake but it showed that soy did not influence NAF volume and estrogen level [72]. Those who have a fruit and vegetable diet showed a decline in NAF production and hormone levels [73]. The dietary intake also showed to affect the micronutrients including carotenoids and soy isoflavones of NAF [74].

**Table 2:** A semi-quantitative analysis of liquid biopsies for breast cancer biomarker scanning

Liquid biopsy	Volume	Accessibility	Discomfort	Reference sample	Sample preparation	Biomarker concentration	Patient-led collection	Convention
Plasma	++	+++	+++	Absent	++++	++	Absent	+++++
Urine	+++++	+++++	+	Absent	+++	+	Present	++++
Saliva	+++	+++++	+	Absent	++	+	Present	+++
Ductal lavage	+	+	++++	Present	+	+++++	Absent	+
Random Periareolar Fine Needle Aspiration	+	+	+++++	Present	++	+++++	Absent	+
Nipple Aspirate Fluid	+	+++	++	Present	+	+++++	Present	++

### NAF biomarkers

The level of testosterone in NAF could be a suitable biomarker for predicting cancer. It is related to different testosterone levels of serum found in premenopausal and post-menopausal women [75]. Another study showed that in women who are at the pre-menopausal stage and have cancer, the level of free testosterone in NAF was higher than albumin-bound testosterone [76]. A separate study on the expression of antigens Thomsen Friedenreich (TFr) and Tn helped discover that NAF secreted from 90% of women with breast cancer had increased concentration of TFr and Tn when differentiated with NAF from healthy women. This was because the two antigens are found on the epithelial cancer cell surface [77]. It was reported that the concentration of both antigens TFr and Tn was low in benign cancer-affected women. Moreover, the expression of TFr showed more precise results in detecting cancer or atypia in contrast to Tn [78]. NAF is highly enriched with proteins especially originating from the ducts lined by epithelium. The concentration of protein in NAF can be up to 170 mg/ml higher than in plasma [79] Some of these proteins are beneficial biomarkers. Kallikrein − 3 (KLK3) or (PSA) prostate-specific antigen is found to be present in breast-related cancer. It was first seen in seminal plasma and prostate tissues secreted by the epithelia lining the ducts of the acini and prostate gland. It was also seen that women who were not affected by breast cancer before showed increased levels of KLK3 while women with invasive cancer showed decreased levels [80]. Superoxide dismutase Cu-Zn (SOD-1) content within NAF showed reduced levels in women having breast tumors. SOD-1 contributed to the initiation of cancer and proliferation damage of reactive species of oxygen. Hence, the conclusion was made that the SOD 1 concentration can help identify normal and cancerous cells, as it could be a major antioxidant enzyme to determine the health of the breast. Antioxidant DJ 1 mRNA oncogene expression in the ductal carcinoma cells was found to increase. At the protein level, the results obtained were opposite and the expression was found to decrease in cancer patients’ blood. NAF analysis on 136 women showed an increased concentration of DJ -1 protein in NAF secreted from patients of cancer. However, their levels were low in benign papillary tumors [81]. Samples were also collected for cytokine profiling but no variation was observed in anti-inflammatory cytokines including IL-4, IL-9, IL-10, IL-13, or in any of the pro-inflammatory cytokines including – IL-2, interferon-gamma, or in immuno-modulatoryinterleukins-IL-5, IL-7 or in chemokines- eotaxin, RANTES, IP-10. The NAF of breast cancer victims having increased aluminum content in the breast showed increased levels of cytokines (pro-inflammatory) which included p70, IL-12, IL-6, and TNF-alpha, while C-C including MIP-1alpha and MCP-1 and the CXC included IL-8. Therefore, this study related the pro-inflammatory IL -6 and chemokines (monocyte chemo-attractant)– MCP-1 and MIP1aplha, aluminum, and oxidative stress in NAF from breast cancer women [82].

Cancer-causing cells have been reported with high amounts of ferritin (FTN) and transferrin (TF) increased expression of trans ferritin receptors which indicates that the metabolism conducting proteins of iron have an essential role in breast cancer development [83]. Therefore, the levels of soluble FTN and TF may help in the early screening of breast cancer. The components of plasma, Plasminogen activator inhibitor inhibits proteases directing while promoting proliferation of breast cancer and metastasis. This shows that increased proteolysis of plasmin may check the gathering of tumor blood vessels, cell adhesion modulation, and stimulate cell proliferation [84]. Hence, it can be concluded that an enormous amount of PAI-1 and uPA in women after menopause while high levels of Thomson Friedenreich antigen in premenopausal women have appeared in NAF of breast cancer victims [85].

Observation of different types of cancer along with advanced stages of breast cancer, C-reactive protein (CRP) is used as a biomarker [86]. High levels of CRP in the ductal epithelial cells of breasts suggest an inflammatory process related to the initial stages. An observation was made on 59 patients that showed CRP in NAF had a positive relation to the Gail model used in the detection of breast cancer chances [87].

**Table 3:** Illustrating different types of Biomarkers along with their characteristics

Biomarker	Characteristics
Prostate-specific antigen (PSA) [76, 80, 88]	Is opposite to the stage of disease, tumor size, node, and distant metastasis.
Thomsen–Friedenreich (TFr) and Tn antigens [77, 78]	Helps to identify cancer or atypia
Testosterone [75, 76]	is effective only in women post menopause
Protein DJ-1 [81]	The mRNA level rises in tissues however the protein level declines
Cytokines/chemokines [82]	CXC and pro-inflammatory C–C chemokines are present at high levels.
Plasminogen activator inhibitor-1 (PAI-1), (uPA) urokinase-type plasminogen activator [84, 85]	Aids cancer initiation and progression
Serotransferrin protein (TF) and (FTN) ferritin [89]	Helps in the progression of cancer
C-reactive protein (CRP) [86, 87]	Serum biomarker detects the spread of a variety of cancer

### NAF proteomics

Characterization of NAF based on proteomics showed immense development in the technology of mass spectrometry and also in the advancement of novel quantitative techniques. The proteomic analysis can be used as a profitable move to analyze and figure out the disease etiology and also for the discovery of biomarkers [88]. Proteomic characterization of NAF specimens taken from healthy and cancerous breasts from surface-enhanced laser desorption ionization mass spectrometry (SELDI-MS) showed zero variations in the SELDI-MS peak [89]. Other separation techniques were used and were identified by ion trap mass spectrometer, out of 64 proteins with depleted immune NAF specimens 15 were earlier found to vary in cells of serum and tumor isolated from breast cancer victims with cathepsin D and osteopontin [90]. 41 different components of NAF were identified through 2D PAGE protein separation and digestion with trypsin and matrix-assisted laser desorption ionization time-of-flight mass spectrometer (MALDI-TOF) [91]. The levels of apolipoprotein-D, prolactin-inducible protein, and alpha-1 acid glycoprotein were seen to vary in the NAF of cancer patients. The ELISA test suggested that the protein expression was connected with premenopausal and postmenopausal cancer. The tumor-specific proteins in NAF were analyzed qualitatively and quantitatively through Isotope-coded affinity tag (ICAT) tandem mass spectrometry (MS). Therein, 39 proteins of NAF were identified amongst 353 peptides isolated from 18 cancer patients. NAF samples collected from diseased persons showed decreased levels of Alpha − 2-HS -glycoprotein, however, the beta–globin, hemopexin, lipophilic B, and vitamin binding protein showed increased levels. Six NAF specimens collected from three fit individuals and three cancer-affected individuals were taken and analyzed by using LTQ -Orbitrap XL mass spectrometer, wherein 854 or even more distinctive proteins were identified along with biomarkers of breast cancer, tissue plasminogen activator, uPA, cancer antigen 15.3, and cathepsin D [92]. Another study identified greater than 1900 gene products having factors with mitotic growth (IGF1, IGF2, VEGFA, PDGFC, PGGFD, EGF) proteins for cell fixation (NCAM2, ICAM1, CEACAMs) with biomarkers of breast cancer (mucin-16/CA -125, mucin-1/CA 15 − 3, EGFR, MUCL1, Cytokeratins 5,8,14,18) and the National Cancer Institute Early Detection Research Network is investigating about 46 candidate biomarkers [93]. About 1200 proteins per sample were found and the similar NAF pairs were compared from a fit individual having DCIS, benign cancer, and invasive carcinoma [94]. The complementary pairs showed strong similarity in their profile, whereas the solo samples showed variations, this was done by the use of SDS PAGE and 2D-DIGE NAF analysis [95]. Constituents in milk proteins in fit individuals were found to be in surplus amounts. The proteins present in milk were found to have galactorrhea-inducing discharge from the nipple [94]. Various profiles of proteins reported within NAF highlighted the ability to identify biomarkers that could help in breast cancer development. However, there are certain limitations of NAF in proteomic analysis. Due to low cell count the analysis of body fluids such as NAF can only be made through proteomics. The 2D PAGE technology used for proteomic analysis though is low throughput and labor involving procedure, it distinguishes and selects only highly abundant proteins, moreover, it selects against the proteins having high acidic or basic nature [100].

## Conclusion

The main reason for mortality in women all around the world is breast cancer. Although many attempts have already been made to figure out the reasons and the latest treatment technologies, the possibility of the occurrence of cancer can only be prevented by surgical interventions. This is due to present methods of regular detection that also have certain limitations. Identifying biomarkers can be effective in the detection of cancer at an acute stage and can provide great relief but it has not been completely achieved to date. The tissues related to cancer are not readily accessible. Moreover, the biomarkers get diluted when secreted from the affected region. Biofluid collected from healthy as well as diseased women can be beneficial to analyze the disease. Additionally, the biomarkers related to NAF have a good ability to develop exceptional, non-invasive patient screening procedures. When liquid biopsy of NAF is made, it provides several benefits to premenopausal women where the current diagnostic process is less, and can often cause the synthesis of NAF in women at a postmenopausal stage wherein the ductal atrophy occurs more commonly. The NAF production causes little or no discomfort in comparison to other breast cancer screening techniques [96, 97]. It helps in getting a similar pair of samples that show an inner control for comparison between healthy and diseased. NAF is secreted in close relation to the cell lining the duct which in turn is related to 85% of breast cancers. The biomarkers are too concentrated for analysis while the preparation of the sample is also decreased when it is brought to comparison with the tissues that need yield-reducing extraction of protein. Although the NAF samples are less the protein concentration is too high and while they are more suitable for analyses of replicas with mass spectrometric procedures.

An observation was performed on similar matches of NAF by the use of multiple reaction monitoring mass spectrometry (MRM MS), variations were seen between the healthy and diseased individuals in terms of prolactin inducible protein. With an MRM MS technique, one can easily build a multiplexed assay which would count for numerous markers for a healthy breast and will indicate a certain component in it that varies from patient to patient. Study on different kinds of biomarkers helps in improving specificity and in the growth of a clinical assay. This can in turn help in the recognition of cancer in women’s breasts at an acute level, which may be useful regularly to check for cancer at an acute level and in avoiding extreme programs like elective bilateral mastectomy. Biomarker analysis in NAF can help display a paradigm shift in the management of breast cancer which can help women patients to self-analyze breast cancer development.

## Abbreviations

NAF: Nipple Aspirate Fluid
ER: Estrogen Receptor
PR: Progesterone Receptor
HER2: Human Epidermal Growth Factor Receptor
TNBC: Triple Negative Breast Cancer
DBT: Digital Breast Tom synthesis
PET: Positron Emission Tomography
MRI: Magnetic Resonance Imaging
DL: Ductal Lavage
RPFNA: Random Peri-areolar fine needle aspiration
